# Temporomandibular joint ganglion-induced external auditory canal obstruction associated with osteoarthritis: a case report

**DOI:** 10.1093/jscr/rjae791

**Published:** 2024-12-17

**Authors:** Nao Yamamoto Nishimura, Takasuke Usuki, Soju Seki, Takahiro Nishio, Miki Ishibashi, Munehiro Hamaguchi

**Affiliations:** Department of Oral and Maxillofacial Surgery, Yao Municipal Hospital, 1-3-1, Ryugecho, Yao-city, Oaka 581-0069, Japan; Department of Cancer Oral Care, Dentistry and Oral Maxillofacial Surgery, Osaka International Cancer Institute, 3-1-69, Ohtemae, Chuo-ku, Osaka 541-8567, Japan; Department of Oral and Maxillofacial Surgery, Yao Municipal Hospital, 1-3-1, Ryugecho, Yao-city, Oaka 581-0069, Japan; Department of Oral and Maxillofacial Surgery, Yao Municipal Hospital, 1-3-1, Ryugecho, Yao-city, Oaka 581-0069, Japan; Department of Oral and Maxillofacial Surgery, Yao Municipal Hospital, 1-3-1, Ryugecho, Yao-city, Oaka 581-0069, Japan; Department of Cancer Oral Care, Dentistry and Oral Maxillofacial Surgery, Osaka International Cancer Institute, 3-1-69, Ohtemae, Chuo-ku, Osaka 541-8567, Japan; Department of Oral and Maxillofacial Surgery, Yao Municipal Hospital, 1-3-1, Ryugecho, Yao-city, Oaka 581-0069, Japan

**Keywords:** ganglion, temporomandibular joint, external auditory canal

## Abstract

Ganglions are pseudocysts that develop from part of the joint capsule or tendon sheath and are filled with synovial fluid. In this report, we describe a case of external auditory canal obstruction caused by a temporomandibular joint (TMJ) ganglion that was thought to be related to osteoarthritis. A 62-year-old man with the chief complaint of swelling of the anterior wall of the left external auditory canal underwent cystectomy at the Department of Otorhinolaryngology. Histopathological examination revealed a ganglion. Three months later, the swelling reappeared; computed tomography revealed an enlarged left temporomandibular head, which prompted the patient to visit our department. Subsequently, the ear canal was completely obstructed due to worsened swelling, and TMJ arthroplasty and ear canalplasty were performed. Postoperatively, the patient recovered well with no recurrence of swelling. Ganglions that arise as masses in the external auditory canal may be accompanied by osteoarthritis.

## Introduction

Ganglions are pseudocysts that develop from a part of the joint capsule or tendon sheath, are filled with synovial fluid, are commonly found in the joint capsule of the extremities, including the wrist joints, and temporomandibular joint (TMJ) ganglions are rare [1]. In this report, we describe a case of obstruction of the external auditory canal caused by a TMJ ganglion, probably due to osteoarthritis.

## Case report

**Figure 1:**
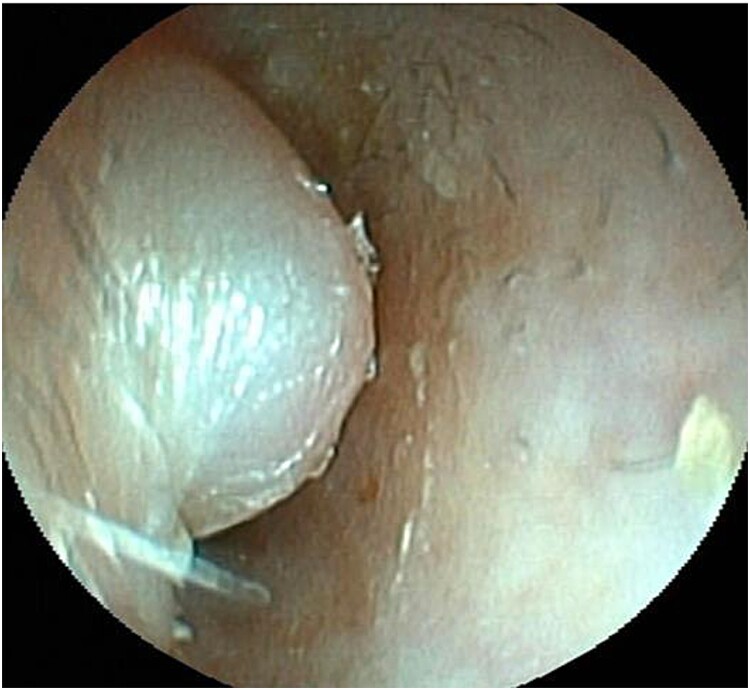
Endoscopic view of the auditory canal. Swelling of the anterior wall of the left lateral auditory canal.

**Figure 2:**
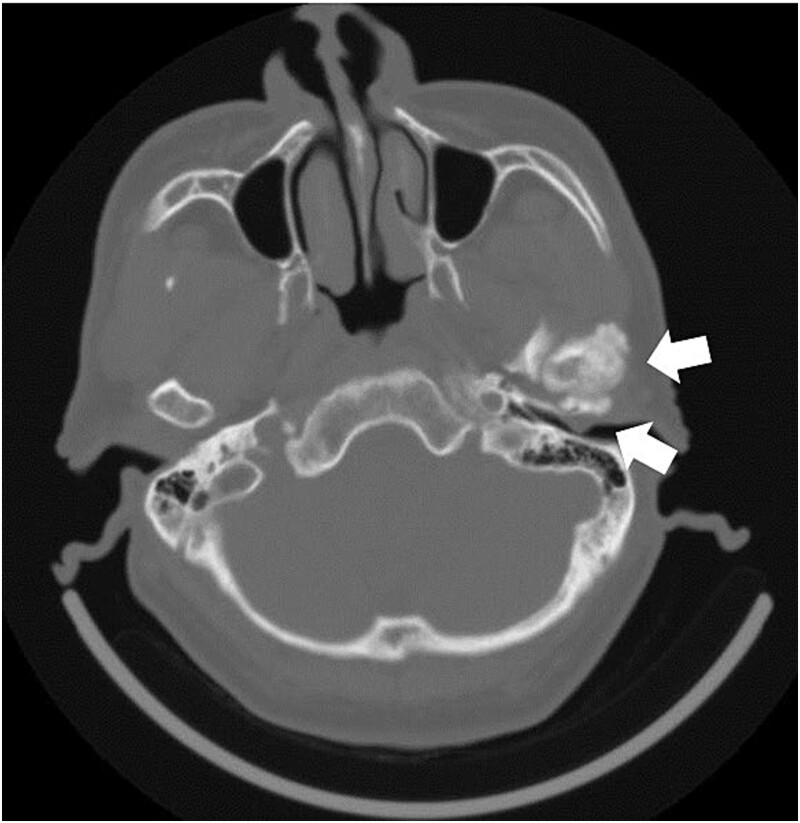
Axial section image of bone conditions on contrast-enhanced CT. CT image shows enlargement of the left temporomandibular head and a mass in the external auditory canal.

**Figure 3:**
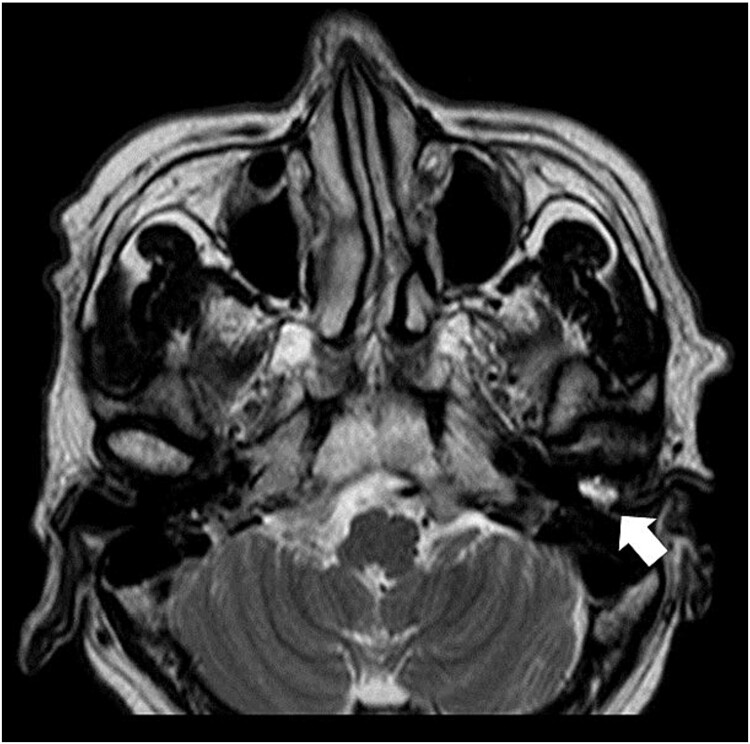
Axial T2-weighted MRI. MRI image shows a high-signal area in the left external auditory canal.

A 62-year-old man presented with a left external auditory canal obstruction and a smooth swelling on the anterior wall. Audiological exams showed no abnormalities, and no otorrhea was observed. Medical history included emphysema, duodenal cancer, and gastric ulcer. The patient was diagnosed with an external auditory canal cyst and underwent cystectomy at the Department of Otorhinolaryngology of our hospital in February 2017. Histopathological examination revealed a cystic lesion without epithelial lining, diagnosed as a ganglion-like lesion. In May of the same year, the swelling was observed again (Fig. 1). The patient was referred to our department for further investigation because computed tomography (CT) revealed enlargement of the left mandibular head (Fig. 2). Clinical findings at the time of consultation included symmetrical facial appearance and a maximum range of motion of 55 mm in the mandible. Upon opening the mouth, crepitus was observed in the left TMJ; however, no restriction of the lateral or anterior movement of the mandible or occlusal deviation was observed. There was no pain in opening the mouth or tenderness in the TMJ. The anterior wall of the left external auditory canal was swollen, with elasticity and no mobility. T2-weighted magnetic resonance imaging revealed an internal high-signal mass in the external auditory canal (Fig. 3).

**Figure 4:**
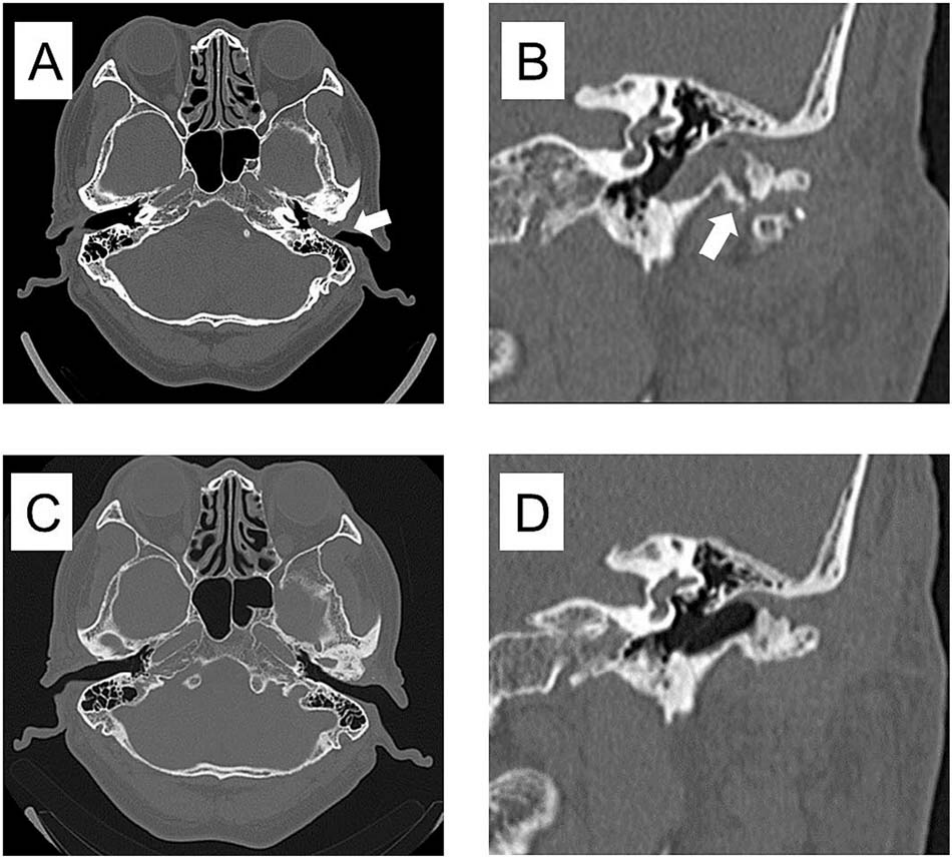
CT imaging findings when the mass was enlarged and the ear canal was obstructed and 3 years after surgery. (A) Axial section CT shows occlusion of the left external auditory canal. (B) Coronal section CT shows a bone defect from the temporomandibular joint to the external auditory canal. (C) Axial section of postoperative 3 years CT shows resolution of the mass in the external auditory canal. (D) Coronal section of postoperative 3 years CT shows restoration of bone defects from the TMJ to the external auditory canal.

**Figure 5:**
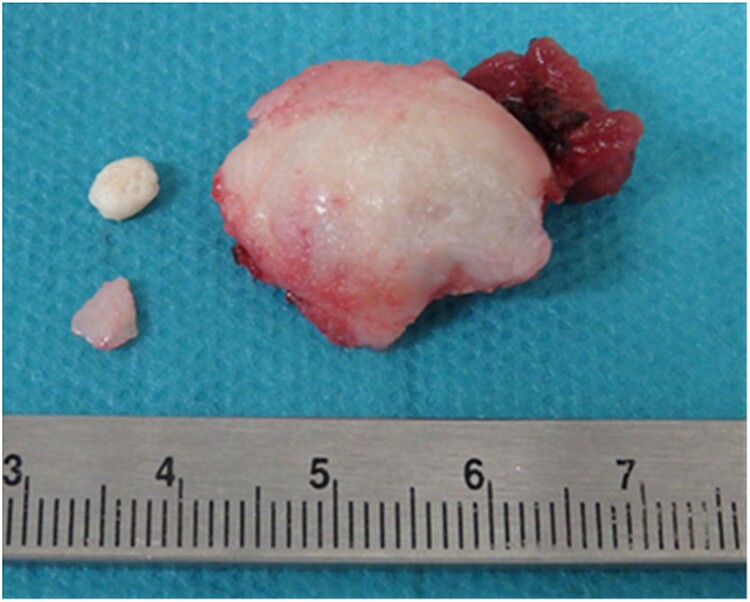
Resected mandibular head and surrounding calcification.

Based on these findings, a clinical diagnosis of TMJ osteoarthritis and TMJ ganglion was made. In July 2017, the mass in the external auditory canal ruptured, and the swelling disappeared; however, the mass flared up again in September, and in October 2017, the left external auditory canal was completely occluded. Axial-section CT showed occlusion of the left external auditory canal, and coronal-section CT showed a bone defect extending from the TMJ to the external auditory canal. (Fig. 4A, B). The patient requested surgery and underwent a left-sided mandibulectomy under general anesthesia and auditory canalplasty performed in December. Intraoperatively, the TMJ was approached via a left preauricular incision, the joint space was opened, and the mandibular head was removed via an 8-mm horizontal osteotomy. During surgery, the surrounding calcified material was removed, as was the anteriorly dislocated articular disc because it was deformed (Fig. 5). Subsequently, transcanal endoscopic ear surgery was performed by otolaryngologist. A skin incision was made before the mass, and a clear jelly-like fluid was found. After the surgery, the patient had intermaxillary fixation for 3 days and began opening training on the fourth day. The opening size improved to 48 mm on the 10th day, and the patient was discharged 13 days after the surgery.

**Figure 6:**
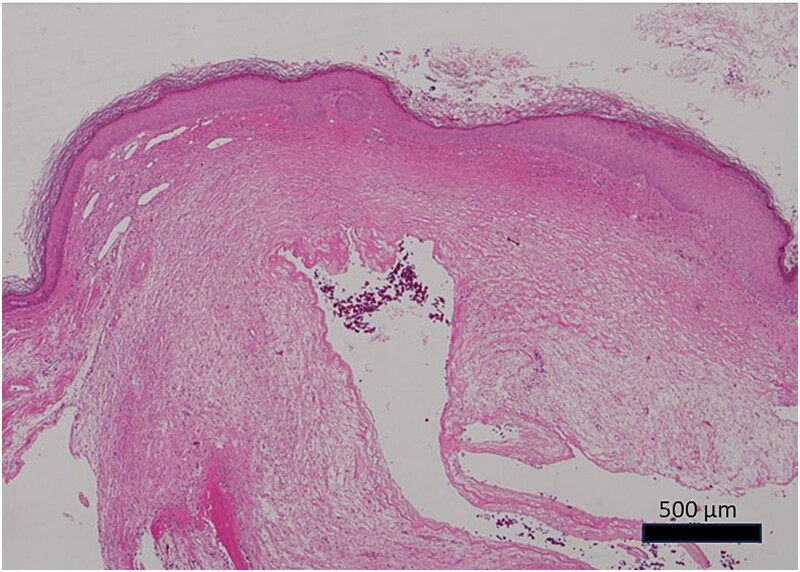
Photomicrograph of the histopathological specimen (HE staining). The cyst had no epithelial covering, mixoid-like changes, and ganglion-like findings.

Histopathological findings showed an edematous subcutaneous lesion in the mucosa of the external auditory canal with a cyst. The cyst was not covered by epithelium and had a ganglion-like appearance (Fig. 6). Osteocytes on the inner surface of the mandibular head showed no tumor-like changes. Still, there were substantial changes in the surface cartilage. This was consistent with osteoarthritis.

Three years postoperatively, the swelling in the left external auditory canal did not recur (Fig. 4C, D), the shape of the resected left temporomandibular head had stabilized, and the left TMJ did not develop any opening disorder, deviation, or pain.

## Discussion

Among TMJ ganglions, the occurrence of a mass in the external auditory canal is rare. In the literature, we found seven cases of TMJ ganglions in the ear canal, including the present case [2–7]. Three of these cases showed TMJ osteoarthritis with enlargement of the mandibular head. Otawara *et al.* [8] reported that 6 of 26 cases of TMJ ganglion reported in the literature had a deformed mandibular head. In cases other than those with mandibular head hypertrophy, the ganglion was located in front of the ear and was accompanied by bone resorption of the mandibular head due to pressure [9–12]. In our case, it was assumed that the cause of the problem in the external auditory canal was the narrowing of the joint space, causing the TMJ to have a hernia-like appearance.

The standard treatment for ganglions is surgical excision. Khachi *et al*. [3] reported that mandibuloplasty and external auditory canalplasty were performed for ganglions in the anterior wall of the external auditory canal associated with osteoarthritis and did not recur. In our case, trans-auricular canal removal of the ganglion resulted in recurrence. Subsequently, mandibuloplasty and external auditory canalplasty were performed. The postoperative course of the mandibuloplasty showed a gradual increase in bone growth postoperatively, and the morphology of the mandibular head settled after 2 years. The results of this study suggest that TMJ osteoarthritis with mandibular head enlargement can affect connective tissue and that TMJ arthroplasty can improve prognosis.

In the present case, CT showed a continuous bony defect and internal shadowing from the TMJ; however, intraoperatively, there was no evidence of communication from the joint cavity or external auditory canal on visual examination. The differential diagnoses of masses in the external auditory canal with bone destruction may include rheumatoid arthritis of the TMJ with perforation of the external auditory canal, pearl tumor in the external auditory canal, and tumors of the TMJ. In our case, after 5 months of follow-up since the initial visit, we concluded that a mandibular tumor was unlikely because there was no change in the deformity of the mandibular head. However, we found a complete obstruction of the external auditory canal due to the mass, and the patient requested surgery, including a histological examination of the mandibular head, so we performed a mandibuloplasty. The patient was diagnosed with TMJ osteoarthritis. TMJ ganglions in the external auditory canal may accompany TMJ osteoarthritis. More case studies are needed.
